# Supplementary material for: Effects of Animal Diet and Processing Methods on the Quality Traits of Dry-Cured Ham Produced from Turopolje Pigs
Source: Animals (Basel). 2024 Jan 17;14(2):286. doi: 10.3390/ani14020286 (PMC10812397; doi:10.3390/ani14020286)
Supplement: Supplementary file 1 [file animals-14-00286-s001.zip › animals-2813177-supplementary.pdf]

Effect of animal’s diet and processing method on quality traits of dry-cured ham produced from Turopolje pigs

Danijel Karolyi, Martin Škrlep, Nives Marušić Radovčić, Zoran Luković, Dubravko Škorput, Krešimir Salajpal, Kristina Kljak and Marjeta Čandek Potokar

Supplementary Data, Tables S1, S2 and S3.

Table S1. Effect of animal's diet and processing method on volatile compounds of biceps femoris muscle of TP dry-cured ham.

Volatile Compounds	Processing (P)		Diet (D)		RMSE	Significance		
	Less smoke	Standard	Acorn	Control		P	D	Interaction
**Aldehydes**								
3-Methylbutanal	0.25	0.40	0.44	0.22	0.187	ns	ns	ns
2-Methylbutanal	0.39	0.39	0.50	0.28	0.121	ns	t	ns
Pentanal	0.63	0.54	0.58	0.59	0.243	ns	ns	ns
Hexanal	9.25	6.33	7.29	8.30	4.06	ns	ns	ns
Heptanal	2.42	2.57	2.26	2.74	1.40	ns	ns	ns
Benzaldehyde	1.91	2.14	2.04	2.01	1.33	ns	ns	ns
Octanal	4.68	4.42	5.24	3.86	1.76	ns	ns	ns
Benzeneacetaldehyde	0.86	1.48	1.72	0.62	1.29	ns	ns	ns
Nonanal	6.87	13.13	13.70	6.29	4.11	t	t	ns
2-Nonenal	0.20	0.36	0.48	0.08	0.321	ns	ns	ns
4-Ethyl-benzaldehyde	0.06	0.11	0.10	0.07	0.061	ns	ns	ns
Decanal	0.51	0.81	0.85	0.47	0.239	ns	t	ns
2,4-Nonadienal	0.32	0.31	0.23	0.40	0.326	ns	ns	ns
2-Decenal	0.27	1.30	0.60	0.97	0.832	ns	ns	ns
Tetradecanal	0.15	0.40	0.18	0.38	0.098	*	*	ns
Hexadecanal	0.19	0.48	0.31	0.37	0.039	*	t	ns
Σ Total	29.00	35.40	36.61	27.79	7.00	ns	ns	ns
**Alcohols**								
1-Pentanol	1.09	1.06	0.72	1.44	1.24	ns	ns	ns
3-Methyl-1-butanol	0.09	0.00	0.00	0.09	0.125	ns	ns	ns
1-Hexanol	0.32	0.53	0.43	0.42	0.351	ns	ns	ns
2-Methyl-4-octanol	0.49	0.00	0.03	0.45	0.641	ns	ns	ns
1-Heptanol	0.78	0.85	0.75	0.89	0.427	ns	ns	ns
1-Octen-3-ol	2.83	2.54	2.63	2.73	0.990	ns	ns	ns
2-Ethyl-1-hexanol	4.30	1.90	4.49	1.70	2.85	ns	ns	ns
Benzylalcohol	2.00	0.26	1.36	0.90	1.73	ns	ns	ns
2-(1-methylethyl)-cyclohexanol	1.27	0.00	0.27	0.99	1.13	ns	ns	ns
Phenylethyl alcohol	2.49	1.79	1.93	2.35	2.35	ns	ns	ns
2,6-Dimethyl-4-heptanol	0.00	0.67	0.15	0.52	0.504	ns	ns	ns
2-Phenoxy-ethanol	0.49	0.00	0.29	0.21	0.466	ns	ns	ns
2-Ethyl-hexanol	4.30	1.89	4.49	1.70	2.85	ns	ns	ns
4-Methyl-1-(1-methylethyl)-3-cyclohexenol	0.51	0.00	0.18	0.32	0.183	*	ns	ns
Σ Total	17.06	9.66	13.41	13.31	5.74	ns	ns	ns
**Aromatic hydrocarbons**								
Benzene	0.31	0.00	0.20	0.11	0.215	ns	ns	ns
1,2-Dimethoxy-benzene	0.68	1.23	1.24	0.68	0.584	ns	ns	ns
Pentyl-benzene	0.46	0.31	0.27	0.51	0.178	ns	ns	ns
3,4-Dimetoxytoluene	0.67	1.28	1.32	0.63	0.782	ns	ns	ns
3,5-Dimetoxytoluene	0.00	0.11	0.03	0.09	0.128	ns	ns	ns
1,2,3-Trimethoxybenzene	0.28	0.87	0.61	0.54	0.315	t	ns	ns
1,2,4-Trimethoxybenzene	0.15	1.20	0.17	0.29	0.184	ns	ns	ns

Table S1. Continuation.

Volatile Compounds	Processing (P)		Diet (D)		RMSE	Significance		
	Less smoke	Standard	Acorn	Control		P	D	Interaction
4-Ethyl-1,2-dimetoxybenzene	0.13	0.93	0.24	0.80	0.828	ns	ns	ns
1,2,3-Trimetoxy-5-methylbenzene	0.15	1.20	0.20	1.15	1.44	ns	ns	ns
Σ Total	2.81	6.26	2.29	4.78	3.53	ns	ns	ns
**Ketones**								
2-Pentanone	0.07	0.00	0.07	0.00	0.097	ns	ns	ns
2-Heptanone	0.65	0.63	0.78	0.50	0.650	ns	ns	ns
1-Octen-3-one	0.90	0.13	0.41	0.61	0.450	t	ns	ns
3-Octen-2-one	0.00	0.07	0.00	0.07	0.105	ns	ns	ns
2-Nonanone	1.08	0.65	0.43	1.30	1.29	ns	ns	ns
1-Phenyl-2-propanone	0.34	0.21	0.34	0.20	0.239	ns	ns	ns
2-Decanone	0.84	0.00	0.14	0.70	0.185	*	*	*
2,3-Dihydro-1H-inden-1-one	0.44	0.43	0.32	0.55	0.239	ns	ns	ns
3-Undecanone	0.00	0.05	0.00	0.05	0.065	ns	ns	ns
3-Octadecanone	0.15	0.13	0.08	0.20	0.140	ns	ns	ns
Dihydro-5-pentyl-2-furanone	0.13	0.49	0.28	0.35	0.368	ns	ns	ns
6,10-Dimethyl-5,9-undecadien-2-one	0.16	0.31	0.28	0.18	0.024	*	*	*
2-Hydroxy-3,4-dimethyl-2-cyclopenten-1-one	0.18	0.00	0.14	0.04	0.206	ns	ns	ns
Σ Total	4.92	3.10	3.27	4.75	1.90	ns	ns	ns
**Phenols**								
2-Methylphenol	0.75	1.83	1.02	1.01	0.538	ns	ns	ns
3-Methylphenol	0.61	0.41	0.35	0.66	0.059	*	*	*
4-Methylphenol	1.59	0.51	1.44	0.66	1.68	ns	ns	ns
2-Methoxyphenol	3.85	6.44	5.88	4.41	2.35	ns	ns	ns
2,4-Dimethylphenol	0.00	0.39	0.30	0.09	0.300	ns	ns	ns
2-Methoxy-3-methylphenol	0.20	0.31	0.24	0.27	0.253	ns	ns	ns
3-Ethylphenol	0.23	0.42	0.39	0.26	0.090	*	ns	ns
2,3-Dimethylphenol	0.67	0.00	0.34	0.33	0.126	*	ns	ns
3,4-Dimethylphenol	0.04	0.12	0.09	0.07	0.132	ns	ns	ns
2-Methoxy-4-methylphenol	1.11	3.28	2.77	1.62	0.821	*	ns	ns
2,6-Dimethoxyphenol	0.14	0.05	0.05	0.14	0.218	ns	ns	ns
4-Ethyl-2-methoxyphenol	0.05	1.24	0.66	0.62	0.120	*	ns	ns
2,6-Dimethoxyphenol	0.14	0.05	0.05	0.14	0.218	ns	ns	ns
Eugenol	0.10	0.87	0.19	0.78	0.734	ns	ns	ns
Σ Total	9.61	16.17	14.32	11.45	2.77	*	ns	ns
**Alkanes and alkenes**								
1,2-Dimethyl-cyclopentane	0.06	0.00	0.00	0.06	0.080	ns	ns	ns
3,4,5-Trimethyl-heptane	2.30	1.52	1.44	2.37	0.711	ns	ns	ns
3-Methyl-heneicosane	0.22	0.03	0.13	0.12	0.099	*	ns	ns
Cyclooctane	2.43	2.21	2.41	2.23	2.04	ns	ns	ns
4-Methyl-1-3-cyclohexene	0.00	0.19	0.00	0.19	0.010	*	*	*
Cyclohexane	0.00	0.06	0.00	0.06	0.081	ns	ns	ns

Table S1. Continuation.

Volatile Compounds	Processing (P)		Diet (D)		RMSE	Significance		
	Less smoke	Standard	Acorn	Control		P	D	Interaction
Tridecane	0.21	0.13	0.21	0.13	0.117	ns	ns	ns
Tetradecane	0.13	0.16	0.10	0.19	0.155	ns	ns	ns
Cyclododecane	0.21	0.49	0.29	0.41	0.108	*	ns	ns
1-Pentadecene	0.00	0.19	0.13	0.06	0.104	t	ns	ns
Pentadecane	0.14	0.22	0.18	0.18	0.044	t	ns	ns
Hexadecane	0.06	0.08	0.07	0.06	0.038	ns	ns	ns
Σ Total	5.73	5.27	4.96	6.05	2.18	ns	ns	ns
**Nitrogen compounds**								
2,6-Dimethylpyrazine	1.40	0.18	0.61	1.00	0.701	t	ns	ns
Methoxy-phenil-oxime	1.66	10.20	6.27	5.60	6.78	ns	ns	ns
2,3,5-Trimethypyrazine	1.55	0.52	1.31	0.76	1.15	ns	ns	ns
Σ Total	4.60	10.91	8.19	7.33	6.57	ns	ns	ns
**Terpenes**								
Alpha-phellandrene	0.66	0.14	0.23	0.57	0.351	ns	ns	ns
Alpha-terpinene	0.00	0.17	0.00	0.17	0.244	ns	ns	ns
4-Carene	0.81	0.00	0.05	0.76	1.08	ns	ns	ns
Limonene	0.55	0.42	0.29	0.67	0.437	ns	ns	ns
Cis-beta terpineol	1.17	0.50	0.81	0.85	0.635	ns	ns	ns
Linalool	3.40	0.14	1.97	1.57	2.78	ns	ns	ns
Sabinene	0.00	3.37	0.13	0.24	0.303	ns	ns	ns
Caryophyllene	0.08	0.10	0.02	0.16	0.170	ns	ns	ns
Σ Total	6.66	1.46	3.50	5.00	2.92	t	ns	ns
**Acids**								
Nonanoic acid	0.00	0.07	0.00	0.07	0.100	ns	ns	ns
Hexadecanoic acid	0.40	0.00	0.28	0.12	0.029	*	*	*
Σ Total	0.40	0.07	0.28	0.19	0.100	*	ns	*

*p≤0.05; t - p<0.10; ns - p>0.05; RMSE – root mean square error

Table S2: Effect of animal's diet and ham processing effect (DxP) interaction on volatile compounds of TP dry-cured hams

Trait	Acorn		Control		RMSE
	Less smoke	Standard	Less smoke	Standard	
2-Decanone	0.28^{b}	0.00^{b}	1.39^{a}	0.00^{b}	0.185
6,10-Dimethyl-5,9-undecadien-2-one	0.15^{b}	0.42^{a}	0.18^{b}	0.19^{b}	0.024
3-Methylphenol	0.31^{b}	0.39^{b}	0.90^{a}	0.42^{b}	0.059
4-Methyl-1-3-cyclohexen	0.00^{b}	0.00^{b}	0.00^{b}	0.38^{a}	0.009
Hexadecanoic acid	0.55^{a}	0.00^{c}	0.25^{b}	0.00^{c}	0.028
Total Acids	0.55^{a}	0.00^{b}	0.25^{ab}	0.14^{b}	0.100

^{abc} - mean values of least squares without common superscript letters significantly differ (p≤0.05); RMSE – root mean square error

Table S3. Hedges' g effect sizes for volatile compounds of biceps femoris muscle of TP dry-cured ham.

Volatile Compounds	Processing (P)	Diet (D)
**Aldehydes**		
3-Methylbutanal	0.64	**-1.06**
2-Methylbutanal	0.00	**-1.03**
Pentanal	-0.31	0.08
Hexanal	-0.68	0.23
Heptanal	0.11	0.36
Benzaldehyde	0.19	-0.04
Octanal	-0.14	-0.64
Benzeneacetaldehyde	0.44	**-1.15**
Nonanal	**0.99**	**-1.07**
2-Nonenal	0.40	**-1.11**
4-Ethyl-benzaldehyde	**0.93**	-0.07
Decanal	**0.81**	-0.54
2,4-Nonadienal	-0.01	0.59
2-Decenal	**1.15**	0.29
Tetradecanal	**1.38**	0.31
Hexadecanal	**5.11**	0.03
Σ Total	0.72	-0.54
**Alcohols**		
1-Pentanol	-0.02	0.60
3-Methyl-1-butanol	-0.62	0.31
1-Hexanol	0.61	-0.03
2-Methyl-4-octanol	-0.68	0.54
1-Heptanol	0.15	0.25
1-Octen-3-ol	-0.22	0.07
2-Ethyl-1-hexanol	-0.67	**-1.71**
Benzylalcohol	**-1.01**	-0.72
2-(1-methylethyl)-cyclohexanol	**-1.01**	0.50
Phenylethyl alcohol	-0.28	0.17
2,6-Dimethyl-4-heptanol	**1.15**	0.43
2-Phenoxy-ethanol	**-1.11**	-0.17
2-Ethyl-hexanol	**-1.74**	0.19
4-Methyl-1-(1-methylethyl)-3-cyclohexenol	**-2.34**	0.15
Σ Total	**-1.33**	-0.02
**Aromatic hydrocarbons**		
Benzene	**-1.39**	-0.18
1,2-Dimethoxy-benzene	**0.83**	-0.62
Pentyl-benzene	-0.60	**0.94**
3,4-Dimetoxytoluene	0.66	-0.70
3,5-Dimetoxytoluene	**0.88**	0.14
1,2,3-Trimethoxybenzene	**1.70**	-0.07
1,2,4-Trimethoxybenzene	0.79	0.25

Table S3. Continuation.

Volatile Compounds	Processing (P)	Diet (D)
4-Ethyl-1,2-dimetoxybenzene	**0.87**	0.51
1,2,3-Trimetoxy-5-methylbenzene	0.65	0.56
Σ Total	**1.02**	0.12
**Ketones**		
2-Pentanone	-0.61	-0.31
2-Heptanone	-0.03	-0.61
1-Octen-3-one	**-1.69**	0.27
3-Octen-2-one	0.61	0.27
2-Nonanone	-0.30	0.66
1-Phenyl-2-propanone	-0.43	-0.50
2-Decanone	**-1.52**	0.63
2,3-Dihydro-1H-inden-1-one	-0.01	0.69
3-Undecanone	0.61	0.17
3-Octadecanone	-0.11	**0.97**
Dihydro-5-pentyl-2-furanone	**1.00**	0.10
6,10-Dimethyl-5,9-undecadien-2-one	**1.33**	-0.15
2-Hydroxy-3,4-dimethyl-2-cyclopenten-1-one	**-0.84**	-0.34
Σ Total	**-0.82**	0.59
**Phenols**		
2-Methylphenol	**1.03**	-0.01
3-Methylphenol	-0.71	**1.14**
4-Methylphenol	-0.65	**-0.88**
2-Methoxyphenol	**1.07**	-0.44
2,4-Dimethylphenol	**1.15**	-0.35
2-Methoxy-3-methylphenol	0.47	0.10
3-Ethylphenol	**1.41**	-0.18
2,3-Dimethylphenol	**-5.62**	-0.004
3,4-Dimethylphenol	0.58	-0.09
2-Methoxy-4-methylphenol	**1.91**	-0.54
2,6-Dimethoxyphenol	-0.36	0.34
4-Ethyl-2-methoxyphenol	**8.02**	-0.01
2,6-Dimethoxyphenol	**5.25**	-0.03
Eugenol	**0.91**	0.61
Σ Total	**2.00**	-0.38
**Alkanes and alkenes**		
1,2-Dimethyl-cyclopentane	-0.61	0.21
3,4,5-Trimethyl-heptane	**-0.80**	**1.38**
3-Methyl-heneicosane	**-1.91**	-0.01
Cyclooctane	-0.11	-0.19
4-Methyl-1-3-cyclohexene	**1.06**	0.41
Cyclohexane	0.61	0.21

Table S3. Continuation.

Volatile Compounds	Processing (P)	Diet (D)
Tridecane	-0.68	-0.41
Tetradecane	0.19	0.53
Cyclododecane	**1.94**	0.13
1-Pentadecene	**1.65**	-0.09
Pentadecane	**1.86**	-0.005
Hexadecane	0.42	-0.06
Σ Total	-0.21	0.54
**Nitrogen compounds**		
2,6-Dimethylpyrazine	**-1.15**	0.40
Methoxy-phenil-oxime	1.34	-0.09
2,3,5-Trimethypyrazine	**-0.89**	-0.61
Σ Total	**1.01**	-0.11
**Terpenes**		
Alpha-phellandrene	**-0.90**	0.53
Alpha-terpinene	0.62	0.46
4-Carene	-0.66	0.56
Limonene	-0.25	**0.94**
Cis-beta terpineol	**-1.14**	0.07
Linalool	**-1.24**	-0.18
Sabinene	**1.22**	0.16
Caryophyllene	0.10	**0.82**
Σ Total	**-1.60**	0.45
**Acids**		
Nonanoic acid	0.61	0.25
Hexadecanoic acid	**-2.75**	-0.15
Σ Total	**-1.82**	-0.13

Large effect sizes (Hedge's g > 0.8) are indicated in bold
